# Acute Phase Proteins in Cats Naturally Infected or Seropositive for *Leishmania infantum*

**DOI:** 10.3390/ani16111625

**Published:** 2026-05-27

**Authors:** Eva Spada, Germano Castelli, Federica Bruno, Fabrizio Vitale, Eugenia Oliveri, Maria Liliana Di Pasquale, Vito Biondi, Antonella Migliazzo, Roberta Perego, Luciana Baggiani, Lora Koenhemsi, Merve Alan, Mehmet Erman Or, Daniela Proverbio

**Affiliations:** 1 Department of Veterinary Medicine and Animal Sciences, University of Milan, 26900 Lodi, Italy; eva.spada@unimi.it (E.S.); roberta.perego@unimi.it (R.P.); luciana.baggiani@unimi.it (L.B.); daniela.proverbio@unimi.it (D.P.); 2 Centro di Referenza Nazionale per le Leishmaniosi, Istituto Zooprofilattico Sperimentale della Sicilia “A. Mirri”, 90129 Palermo, Italy; fabrizio.vitale@izssicilia.it (F.V.); eugenia.oliveri@izssicilia.it (E.O.); marialiliana.dipasquale@izssicilia.it (M.L.D.P.); 3 Department of Veterinary Sciences, University of Messina, 98168 Messina, Italy; vito.biondi@unime.it; 4 Dipartimento di Prevenzione UOC Sanità Animale, Igiene degli Allevamenti e delle Produzioni Zootecniche, ASL Latina, 04100 Latina, Italy; a.migliazzo@ausl.latina.it; 5 Internal Medicine Department, Faculty of Veterinary Medicine, İstanbul University-Cerrahpaşa, 34320 Istanbul, Türkiye; lorakoenhemsi@iuc.edu.tr (L.K.); ermanor@iuc.edu.tr (M.E.O.)

**Keywords:** feline leishmaniosis, *Leishmania infantum*, acute phase proteins, serum amyloid A, haptoglobin, ceruloplasmin, serum protein electrophoresis, dysproteinemia

## Abstract

Feline leishmaniosis (FeL) caused by *Leishmania infantum* is increasingly recognized in endemic areas, but the inflammatory profile during infection remains poorly defined. This case–control study evaluated the acute phase proteins (APPs) serum amyloid A (SAA), haptoglobin (Hp), and ceruloplasmin (Cp) in cats with anti-*L. infantum* antibodies and/or DNA and compared them with healthy negative controls. A total of 125 cats were enrolled, including 62 cats positive by IFAT and/or qPCR and 63 healthy controls. Total protein, albumin, albumin-to-globulin ratio (A/G), and serum protein electrophoresis were also assessed, and correlations with APPs were investigated in positive cats. Compared with controls, *L. infantum*-positive cats had significantly higher Hp and Cp concentrations, whereas SAA did not differ significantly. They also showed higher total protein, beta2- and gamma-globulin concentrations and lower albumin and A/G ratio. SAA and Hp were negatively correlated with albumin, whereas Cp was positively correlated with total protein and beta-globulin fractions. No significant correlations were found between APPs and IFAT antibody titer. In exploratory analyses, IFAT seropositivity (cut-off ≥ 1:80) was associated with higher Hp, whereas qPCR positivity was associated with higher SAA and Cp concentrations; however, these results should be interpreted cautiously because qPCR-positive cats were few. These findings indicate a measurable inflammatory and dysproteinemic profile in cats with laboratory evidence of *L. infantum* exposure and/or infection, but do not establish APPs as disease-specific diagnostic markers. However, diagnostic heterogeneity, limited qPCR testing, lack of standardized clinical staging, and possible confounding diseases limit causal and clinical interpretation.

## Simple Summary

*Leishmania infantum* infection and exposure in cats are increasingly recognized in endemic areas, but data on the associated inflammatory response remain limited. Acute phase proteins (APPs) are markers of inflammation, although their role in cats with *L. infantum* infection or seropositivity is still uncertain. In this study, we evaluated serum amyloid A (SAA), haptoglobin (Hp), and ceruloplasmin (Cp) in cats with anti-*L. infantum* antibodies and/or parasite DNA and compared them with healthy negative controls. Overall, cats positive for *L. infantum* antibodies and/or DNA showed higher Hp and Cp concentrations than controls, while SAA did not differ significantly between groups. In exploratory analyses, IFAT seropositivity was associated with higher Hp concentrations, whereas qPCR positivity was associated with higher SAA and Cp concentrations; however, these subgroup findings should be interpreted cautiously because few cats were qPCR-positive, and most seropositive cats had low antibody titers. Positive cats also showed a protein profile consistent with chronic inflammatory stimulation, including higher total protein and globulin fractions and a lower albumin-to-globulin ratio. These findings describe a measurable inflammatory and dysproteinemic profile in cats with laboratory evidence of *L. infantum* exposure and/or infection, but do not establish APPs as disease-specific diagnostic markers.

## 1. Introduction

*Leishmania infantum* is a vector-borne protozoan of major veterinary and zoonotic importance in endemic areas, particularly in the Mediterranean basin. Although the dog is regarded as the main domestic reservoir, cats can also become infected and may develop feline leishmaniosis (FeL), a condition whose clinical expression ranges from subclinical infection to overt systemic disease [1,2]. Current evidence indicates that the most frequent clinicopathological abnormalities reported in infected cats include hyperproteinemia, hypergammaglobulinemia, and abnormalities in serum protein electrophoresis, although the spectrum of findings is variable and still incompletely defined [3,4,5].

The diagnosis of feline *L. infantum* infection remains challenging. No single test can be considered a universal gold standard, and the diagnostic approach usually relies on a combination of serological and molecular methods interpreted together with clinical and clinicopathological findings. Among serological tests, the indirect fluorescent antibody test (IFAT) is one of the most used methods in cats, while polymerase chain reaction (PCR) is employed for direct detection of parasite DNA in blood or tissues. Previous studies have shown that serology and PCR provide complementary rather than interchangeable information, and that antibody positivity may occur in both clinically healthy and sick cats [3,6,7]. In cats, IFAT positivity should be interpreted as serological evidence of exposure or humoral response, whereas PCR positivity indicates detection of parasite DNA.

Acute phase proteins (APPs) are important biomarkers of inflammation in cats. Serum amyloid A (SAA) is considered the major positive APP (10- to 100-fold increase in response to inflammation) and one of the most responsive APPs in cats. Haptoglobin (Hp) is a moderate APP (five- to 10-fold increase), and ceruloplasmin (Cp) is a minor APP (two-fold increase), whereas albumin behaves as a negative APP [8,9,10]. In feline medicine, APPs have been investigated in several infectious and inflammatory disorders and may contribute to the assessment of inflammatory activity and disease monitoring [9,10,11].

Current evidence suggests that the acute phase response in cats with *L. infantum* infection is still only partially characterized. In FeL, the literature is scarce, and the best direct evidence currently available concerns SAA and serum protein electrophoresis rather than a full APP panel. In a study specifically addressing this issue, cats seropositive and/or PCR-positive for *L. infantum* had a significantly higher frequency of increased alpha2 and gamma globulins than healthy controls, whereas SAA concentrations did not differ significantly from those of healthy cats; importantly, these abnormalities were also seen in cats with other inflammatory or neoplastic diseases, limiting their specificity for FeL [12]. Therefore, at present, APP alterations in infected cats seem to indicate the presence of inflammation, but they cannot yet be considered disease-specific markers of *L. infantum* infection. Consequently, the rationale for evaluating APPs in this context is not to establish a diagnostic test for FeL, but to better describe the inflammatory phenotype of cats with different laboratory evidence of exposure and/or infection.

As regards the serum protein profile, the most consistent finding reported in cats with clinical or subclinical *L. infantum* infection is dysproteinemia characterized by hypergammaglobulinemia, usually polyclonal, often associated with increased alpha2-globulins and sometimes with hyperproteinemia and hypoalbuminemia. In particular, hypergammaglobulinemia is one of the most common clinicopathological abnormalities in FeL, alongside mild nonregenerative anemia in some cases [1,2,3,4,5,12,13,14,15]. Overall, the current state of knowledge indicates that the protidemic profile of cats seropositive or infected with *L. infantum* is more consistently altered than their measurable acute phase response: the electrophoretic pattern often suggests chronic antigenic stimulation, whereas evidence for clear increases in individual APPs such as SAA, Hp, or Cp remains limited or still insufficient in this species and disease context. In addition, serum protein electrophoresis in FeL may support the diagnostic interpretation when evaluated together with IFAT and PCR results. Because APPs are useful but non-specific biomarkers of systemic inflammation in cats [8,9,10], their evaluation may provide additional information in feline leishmaniosis, a disease that remains incompletely characterized and diagnostically challenging [1,2]. However, data on APP concentrations in cats exposed to or infected by *L. infantum* are still scarce and mainly limited to SAA and serum protein electrophoresis [5,12]. Therefore, the aim of this study was to evaluate SAA, Hp, and Cp concentrations in cats naturally exposed to or infected by *L. infantum* and to explore their relationship with serological and molecular diagnostic results.

## 2. Materials and Methods

### 2.1. Study Design and Animals

This observational case–control study included serum samples from cats with laboratory evidence of exposure to and/or infection with *L. infantum* evaluated at the Veterinary Transfusion Research Laboratory (REVLab), Department of Veterinary Medicine and Animal Sciences (DIVAS), University of Milan, Lodi (LO), Italy, between October 2020 and June 2023. Cases and controls were enrolled based on the availability of serum samples and anamnestic and signalment data.

Serum samples of FeL cases were from a previous study in which 124 cats seropositive or infected by *L. infantum* were identified [16]. From this population, 21 cats were excluded because they were retrovirus seropositive, and 64 because of insufficient serum samples. In addition to these samples, 23 cats were identified from a population of Turkish cats from Istanbul, for a total of 62 FeL cases. Molecular testing was performed only when a suitable anticoagulated blood or lymph node sample was available from the previous diagnostic work-up or from the Istanbul cohort; therefore, real-time PCR (qPCR) testing was not performed systematically in all cats. The Istanbul cats were sampled from cats evaluated at the Veterinary Medicine Faculty, Internal Medicine Department, Istanbul University-Cerrahpasa, in the Istanbul metropolitan area.

Cats were classified as positive based on laboratory evidence of exposure and/or infection, namely IFAT seropositivity and/or qPCR positivity. The diagnostic criteria adopted for the *L. infantum*-positive group were anti-L. infantum IgG by IFAT at a titer ≥ 1:80 and/or qPCR detection of *L. infantum* DNA in blood or lymph node. Cats were excluded when serum volume was insufficient for APP and protein analyses, or when they were seropositive for feline immunodeficiency virus (FIV) antibody and/or feline leukemia virus (FeLV) antigen (SNAP Combo Plus; IDEXX Laboratories, Westbrook, ME, USA). Detailed clinical staging was not available in a standardized form for all positive cats; therefore, clinical status was not used to define subgroups.

A total of 63 control cats were evaluated for diagnostic purposes at the University of Milan Veterinary Teaching Hospital, Italy. These cats underwent clinical examination and standard hematological and biochemical profiles before neutering surgery, or in cats presented for routine health screening examinations. All were seronegative for FIV antibodies and FeLV antigens and tested negative for *L. infantum* by IFAT and qPCR on blood.

For each cat, the following data were recorded when available: age (kitten < 1 year, young adult 1–6 years, mature adult 7–10 years, senior > 10 years) [17], gender (male or female) and reproductive status (neutered or intact), breed (domestic shorthair–DSH and longhair–DLH-or other breed), lifestyle (stray colony, shelter, or privately owned cats), origin (north, central and south Italy or Turkey).

For both the *L. infantum*-positive and control groups, total serum protein, albumin, protein electrophoresis fractions, albumin-to-globulin ratio (A/G), and concentrations of SAA, Hp, and Cp were measured.

### 2.2. Sample Collection and Laboratory Analyses

Blood samples were collected by jugular or cephalic venipuncture. Samples for serum analyses were allowed to clot and then centrifuged at 1600× *g* for 10 min, and the obtained serum was analyzed immediately or stored at −20 °C until batch analysis.

Total serum protein levels were quantified by spectrophotometry using the biuret colorimetric assay with reagents supplied by Hagen Diagnostica Srl (Florence, Italy) on a Cobas Mira Classics Plus automated chemistry analyzer (Real Time Diagnostic System Srl, Florence, Italy). Serum protein electrophoresis was then performed to separate albumin and globulin fractions, using the Hydragel Kit β1-β2 on a semiautomated agarose gel electrophoresis platform (Hydrasys, Sebia PN 1210, Issy-les-Moulineaux, France). Electrophoretic curves were processed with Phoresis software for Windows 2000 or XP Pro (Sebia PN 1210), which provided the percentage distribution of each fraction based on optical absorbance. Absolute values, expressed per dL, were calculated automatically from the corresponding total serum protein concentration. The evaluated fractions were albumin, alpha1-, alpha2-, beta1-, beta2-, and gamma-globulins, and the A/Gratio was subsequently derived.

### 2.3. Acute Phase Protein Measurement

The serum concentrations of the investigated APPs were measured according to the manufacturer’s instructions on a COBAS Mira Plus Chemistry Analyser (Real Time Diagnostic Systems Srl, Florence, Italy) using a quantitative commercially available human SAA turbidimetric immunoassay (EUROLyser Diagnostica, Salzburg, Austria; normal value < 10 µg/mL) validated for feline SAA determination [18]. Hp (Hagen Diagnostica Srl, Florence, Italy) and Cp (Biochemical Enterprise Srl—BEN, Milan, Italy) were measured by a previously used [11] human quantitative turbidimetric immunoassay not validated in cats, for which definitive method-specific feline reference intervals were not available.

These analyses were performed at REVLab, and quality control procedures were applied in accordance with routine laboratory standards. Specifically, for all three assays, a calibration curve with five standard points for Hp and Cp and six standard points for SAA was performed, and two calibration-control specimens were analyzed before evaluation of the study samples—one with low and one with high values for each APP—with all results within the manufacturer’s recommended reportable range. For both Hp and Cp, the intra-assay coefficients of variation were calculated on 20 replicate samples, each with low, medium, and high concentrations of each APP, with results of 3.3%, 3.1%, and 5.1% for Hp and 1.7%, 2.5%, and 10.5% for Cp, respectively [11].

### 2.4. IndirectFluorescence Antibody Test (IFAT)

Detection of specific anti-*L. infantum* IgG antibodies was performed by IFAT according to the OIE Terrestrial Manual protocol for leishmaniosis [19]. Briefly, *Leishmania* promastigotes from the WHO reference strain MHOM/IT/80/IPT1 were used as antigen and fixed onto multispot microscope slides (Bio-Mérieux, Marcy L’Etoile, France) using acetone. Cat serum samples were serially diluted two-fold, from 1:40 to 1:5120, in phosphate-buffered saline (PBS; pH 7.2) and subsequently applied to the antigen-coated wells. Slides were incubated at 37 °C for 30 min, and both positive and negative controls were included in each analytical run. A fluorescein-conjugated goat anti-cat IgG antibody (Anti-Cat IgG whole molecule–FITC; Sigma Aldrich, St. Louis, MO, USA) diluted 1:200 in PBS was used as a secondary antibody. The slides were examined under a Leica DM 4000B fluorescence microscope (Leica, Heerbrugg, Switzerland). Samples showing an antibody cut-off titer of ≥1:80 were considered positive [1,6].

### 2.5. Real-Time PCR (qPCR)

DNA was isolated from EDTA whole blood using the PureLink™ Genomic DNA Mini Kit (Thermo Fisher Scientific K182002, Waltham, MA, USA), according to the manufacturer’s protocol. Real-time PCR was performed on a QuantStudio 3 system (Life Technologies, Waltham, MA, USA), following a previously described procedure [20]. Each qPCR reaction was carried out in a final volume of 20 µL, including 10 µL of SsoAdvanced Universal Probes Supermix (Bio-Rad, Hercules, CA, USA), 0.25 µM QLeish probe, 0.3 µM of each primer, and 2 µL of extracted DNA at a concentration of 10 ng/µL. Quantification was based on a standard curve generated from 10-fold serial dilutions of *L. infantum* parasite DNA, corresponding to concentrations ranging from 1 × 10^6^ to 1 parasite/mL. The amplification protocol consisted of an initial denaturation step at 95 °C for 10 min, followed by 40 cycles of denaturation at 95 °C for 15 s and annealing/extension at 60 °C for 35 s.

### 2.6. Statistical Analysis

Statistical analyses were performed using MedCalc^®^ Statistical Software version 22.009 (MedCalc Software Ltd., Ostend, Belgium). The distribution of continuous variables was assessed by the D’Agostino-Pearson test. Descriptive statistics were expressed as mean ± SD for normally distributed variables and median and interquartile range (IQR) for non-normally distributed variables. Categorical data were expressed as absolute numbers and percentages. Differences between FeL cats and controls were evaluated using Student’s *t*-test for parametric data and the Mann–Whitney U test for non-parametric data. Categorical variables were compared using the chi-square test or Fisher’s exact test, as appropriate. Correlations between APP concentrations and clinicopathological variables were evaluated using Spearman’s rank correlation test. In addition, an exploratory subgroup analysis was performed according to the diagnostic test yielding positivity for *L. infantum* infection. APP concentrations were compared between IFAT-positive and IFAT-negative cats and between qPCR-positive and qPCR-negative cats using the Mann–Whitney U test. In cats with both IFAT and qPCR results available, APP concentrations were further compared among four diagnostic subgroups (IFAT-negative/qPCR-negative, IFAT-positive/qPCR-negative, IFAT-negative/qPCR-positive, and IFAT-positive/qPCR-positive) using the Kruskal–Wallis test with Dunn’s post hoc test and correction for multiple comparisons. Statistical significance was set at *p* < 0.05 for all tests. Because qPCR-positive cats were few and qPCR was not available for all cats, analyses based on qPCR status were considered exploratory.

## 3. Results

### 3.1. Population Characteristic

A total of 125 cats were enrolled, including 62 cats positive for *L. infantum* IgG and/or DNA and 63 healthy controls. Exact age was available for 42 *L. infantum*-positive cats and 62 healthy control cats. Median age did not differ significantly between positive cats and controls (2.65, IQR 1.0–5 years, vs. 2.00, IQR 0.83–5 years, *p* = 0.401). No significant differences were found for lifestyle, breed, gender, or reproductive status between cases and controls. However, the age-class distribution differed significantly between groups, with a significantly higher proportion of young adults and a lower proportion of kittens and seniors among *L. infantum*-positive cats. Cases were geographically heterogeneous, whereas all controls were from Lombardy, northern Italy (Table 1).

Diagnostic results for *L. infantum* are summarized in Table 2. Briefly, among the 62 cats positive for *L. infantum* antibodies and/or DNA, all cats were tested by IFAT, whereas qPCR was performed in 34/62 cats (22 blood samples and 12 lymph node samples). Most positive cats were IFAT-positive only (53/62, 85.5%), and most IFAT-seropositive cats had a titer of 1:80 (44/55, 80.0%). Nine cats were qPCR-positive; parasite burden was low to moderate [median 15 Leishmania/mL, range 5–250; IQR 13–65] [20].

### 3.2. Acute Phase Proteins, Total Protein and Protein Fractions

**Figure 1:**
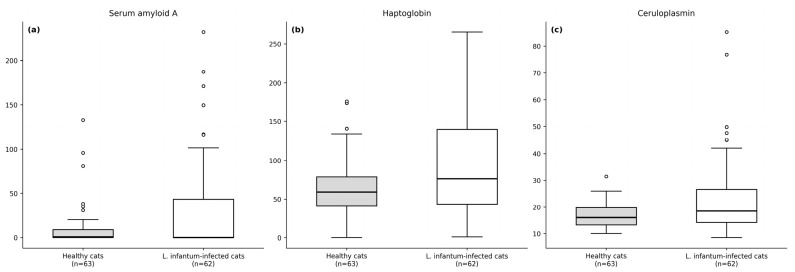
Box-and-whisker plots showing the distribution of serum amyloid A ((**a**), µg/mL), haptoglobin ((**b**), mg/dL), and ceruloplasmin ((**c**), mg/dL) in healthy cats and cats positive for *L. infantum* IgG and/or DNA. Central lines indicate medians, boxes indicate the interquartile range (IQR), whiskers extend to 1.5 × IQR, and points beyond whiskers are plotted as outliers.

When APPs were compared between groups, cats positive for *L. infantum* IgG and/or DNA had significantly higher Hp [76.29 mg/dL (IQR 43.59–139.92) vs. 58.88 mg/dL (IQR 41.17–78.81); *p* = 0.013] and Cp [18.54 mg/dL (IQR 14.32–26.50) vs. 16.13 mg/dL (IQR 13.39–19.86); *p* = 0.012] concentrations than healthy controls. In contrast, SAA did not differ significantly between cats positive for *L. infantum* IgG and/or DNA and controls [0.00 µg/mL (IQR 0.00–43.54) vs. 0.63 µg/mL (IQR 0.00–8.84); *p* = 0.187] (Table 3, Figure 1).

Exploratory analyses according to diagnostic test results showed that IFAT-seropositive cats had significantly higher Hp concentrations than IFAT-negative cats (*p* = 0.022), whereas SAA and Cp did not differ significantly according to IFAT serostatus (*p* = 0.733 and *p* = 0.093, respectively). By contrast, qPCR-positive cats had significantly higher SAA (*p* = 0.001) and Cp (*p* = 0.004) concentrations than qPCR-negative cats, while Hp did not differ significantly (*p* = 0.155) (Table 4).

When cats with both IFAT and qPCR results available were grouped as IFAT-negative/qPCR-negative (n = 62), IFAT-positive/qPCR-negative (n = 14), IFAT-negative/qPCR-positive (n = 7), and IFAT-positive/qPCR-positive (n = 2), overall differences were significant for SAA (*p* = 0.011) and Cp (*p* = 0.001), but not for Hp (*p* = 0.488) (Table 4). In these exploratory subgroup analyses, the highest median SAA and Cp concentrations were observed in qPCR-positive cats, with the greatest values recorded in the IFAT-positive/qPCR-positive subgroup. However, Dunn’s post hoc pairwise comparisons did not remain statistically significant after correction for multiple testing, likely reflecting the small number of qPCR-positive cats and, in particular, the very limited size of the double-positive subgroup.

Compared with controls, cats positive for *L. infantum* IgG and/or DNA had significantly higher total protein, beta2- and gamma-globulin concentrations and lower A/G ratios and albumin concentrations. No significant between-group differences were found for alpha1, alpha2, or beta1-globulin concentrations (Table 3).

In cats positive for *L. infantum* IgG and/or DNA, SAA showed a significant negative correlation with albumin and a significant positive correlation with alpha2-globulin. Hp showed a significant negative correlation with the A/G ratio and albumin and a significant positive correlation with alpha2-globulin. Cp showed a significant positive correlation with total protein, beta1-globulin, and beta2-globulin, and a significant negative correlation with the A/G ratio (Table 5).

No significant correlations were found between any APP and IFAT antibody titre. Quantitative qPCR data were available for too few *L. infantum*-positive cats (blood qPCR, n = 4; lymph node qPCR, n = 5) to support robust correlation analyses. In addition, standardized clinical staging data were not available for these cats, preventing assessment of relationships between APP concentrations and clinical severity.

## 4. Discussion

The present study investigated the behavior of selected APPs in cats positive for antibodies and/or genomic evidence of *Leishmania infantum*. Because this group included most cats positive only by IFAT, many with low titers, the findings should be interpreted as referring to a laboratory-defined *L. infantum*-positive population rather than to confirmed clinically active FeL. The main findings were that Hp and Cp concentrations were significantly higher in *L. infantum*-positive cats than in controls, whereas SAA did not differ significantly between groups. In addition, positive cats showed a protein profile characterized by higher total protein, lower albumin and A/G ratio, and increased beta2- and gamma-globulin fractions. Exploratory analyses according to diagnostic profile indicated associations between IFAT seropositivity and higher Hp and between qPCR positivity and higher SAA and Cp, but these results should be considered hypothesis-generating because of the small number of qPCR-positive cats and the lack of standardized clinical staging.

The lack of a significant increase in SAA in the overall comparison between FeL-positive cats and controls is one of the most interesting results of this study. In cats, SAA is considered the major APP and usually shows marked increases in the presence of acute and intense inflammation [8,9,10]. By contrast, FeL often appears to be characterized by chronic, fluctuating, or subclinical inflammatory stimulation rather than by an acute systemic inflammatory burst [1,2,3,21]. This may partly explain why, in our population, median SAA concentration remained low in the overall case–control comparison despite broad individual variability and the presence of some markedly increased values. At the same time, the exploratory diagnostic subgroup analysis showed significantly higher SAA concentrations in qPCR-positive cats than in qPCR-negative cats, suggesting that SAA may increase preferentially in cats with detectable parasite DNA and possibly greater inflammatory stimulation. Thus, SAA may have limited discriminatory value when FeL-positive cats with heterogeneous clinical and biological status are grouped but may still reflect inflammatory activity in a subset of cats, particularly those with direct molecular evidence of infection. This interpretation is partially in line with previous studies on *L. infantum* infection in cats. Savioli et al. [12] reported that cats exposed to and infected with *L. infantum* showed alterations in SAA together with alpha2- and gamma-globulin changes on serum protein electrophoresis, suggesting that SAA may contribute to the inflammatory profile associated with infection. More recently, Donato et al. [13] described abnormalities in several markers of inflammation in cats tested for *L. infantum* and/or FIV antibodies, further supporting the concept that inflammatory biomarkers can be altered in this infectious context. However, qPCR positivity demonstrates the presence of parasite DNA and should not be interpreted as a direct measure of clinical disease activity.

In contrast, Hp and Cp were significantly higher in FeL-positive cats. This finding is biologically plausible because Hp and Cp generally have a slower and more sustained increase than SAA and may therefore better reflect persistent inflammatory stimulation [8,9,10]. A chronic inflammatory response is compatible with the pathogenesis of FeL, which is driven by prolonged host–parasite interaction and variable immune activation [1,2,3,21]. Interestingly, the exploratory analyses suggested a partially different behavior of these APPs according to diagnostic profile: Hp was higher in IFAT-seropositive cats, whereas SAA and Cp were higher in qPCR-positive cats. Although these observations should be interpreted cautiously due to the limited number of samples, they may suggest that Hp better reflects a more sustained immune-inflammatory response associated with seroreactivity, whereas SAA and Cp may be more responsive in cats with direct molecular evidence of infection. In this respect, our results indicate that different APPs may capture different biological aspects of feline *L. infantum* infection. In addition, these findings support maintaining a distinction between seropositive-only, qPCR-positive-only, and double-positive cats when interpreting diagnostic and inflammatory data. Therefore, these subgroup observations should be considered exploratory and should not be used to infer diagnostic performance or clinical staging.

The protein abnormalities observed in FeL-positive cats are also consistent with previous reports. Hyperproteinemia, hypoalbuminemia, or a reduced A/G ratio, and hypergammaglobulinemia have already been described in cats infected with *L. infantum*, including populations from Italy and Spain [3,4,5,14]. Likewise, serum protein electrophoresis studies in infected but apparently healthy cats have shown abnormalities of the globulin fractions, including polyclonal gammopathy [14]. In a comprehensive review and statistical analysis of clinical management of FeL, Garcia-Torres et al. (2022) [15] reported that hyperproteinemia was the most frequent laboratory abnormality (46.3%), while hypergammaglobulinemia was detected in 71.0% of cases undergoing serum protein electrophoresis, further corroborating the findings of the present study. In our study, the increase in beta2- and gamma-globulins together with the decrease in albumin and the A/G ratio fits well with chronic antigenic stimulation and inflammatory dysproteinemia.

Correlation analysis within FeL-positive cats further supports this interpretation. SAA and Hp were both associated with lower albumin and higher alpha2-globulin concentrations, while Cp was associated with higher total protein, beta1- and beta2-globulin fractions and a lower A/G ratio. These relationships are biologically coherent with the feline acute phase response, in which positive APPs and inflammatory globulin fractions increase, whereas albumin decreases as a negative APP [9,10]. The correlations observed here indicate that APP changes in FeL-positive cats are not isolated events, but part of a broader inflammatory protein response involving the electrophoretic profile.

Interestingly, none of the APPs was significantly correlated with the IFAT antibody titer. This suggests that the magnitude of the antibody response does not necessarily parallel the intensity of systemic inflammatory activation. This is not unexpected, because serology mainly reflects humoral exposure and immune recognition, whereas APPs are markers of inflammatory activity. Previous studies have already highlighted the complexity of interpreting serological results in FeL and the need to combine serology with molecular, clinical, and clinicopathological data [3,6,7]. In our study, the finding that qPCR positivity was associated with higher SAA and Cp concentrations supports the hypothesis that cats with detectable parasite DNA may have a more evident inflammatory response in this cohort. Furthermore, in the four-group exploratory analysis, overall differences in SAA and Cp were significant, and the highest median values were observed in qPCR-positive cats, particularly those positive by both IFAT and qPCR, although post hoc pairwise comparisons did not remain significant after correction for multiple testing. Therefore, in FeL, IFAT, qPCR, APPs, and electrophoresis should be considered complementary rather than overlapping indicators. 

A limitation of this study is that diseases other than retroviral infections were not systematically excluded. This is important because feline APPs are non-specific inflammatory markers and may be altered by several concurrent conditions, including surgery [22], trauma [11], neoplasia [23], oral inflammatory disease [24], and other systemic disorders [10]. Therefore, the APP changes observed in this study cannot be interpreted as exclusively related to *L. infantum* infection. On the other hand, it is important to remember that FeL is frequently associated with comorbidities that may favor the establishment or clinical expression of infection [15]. In addition, the clinical aspects of *L. infantum* infection were not investigated, and the FeL-positive group included cats with potentially different clinical stages; this heterogeneity may have particularly influenced SAA results, and the relationship between APP concentrations and clinical manifestations, disease severity, or outcome could not be assessed. Another important limitation is the limited availability of PCR results and the very low number of qPCR-positive cats, preventing a robust assessment of the association between inflammatory biomarkers and parasite burden. These limitations should be considered when interpreting the present findings, particularly because most *L. infantum*-positive cats were classified based on serology alone, and many had low IFAT titers, which may indicate exposure or humoral response rather than active clinical disease or infection. Moreover, cases originated from geographically heterogeneous regions, including Sicily, Lazio, Lombardy, and Istanbul, whereas controls were all from northern Italy; population, environmental, and epidemiological differences may therefore have influenced the biomarker profile. Finally, qPCR testing was performed in only a subset of cats and on different sample types. This is relevant because diagnostic sensitivity may differ between blood and lymph node samples, with lymph node testing potentially detecting tissue-associated infection more reliably than blood, especially in cases with low circulating parasite loads [1]. For these reasons, the subgroup results according to qPCR status must be considered hypothesis-generating only.

Despite these limitations, the present study adds information on the inflammatory profile of cats with laboratory evidence of *L. infantum* exposure and/or infection. The data suggest a tendency toward a chronic inflammatory and dysproteinemic pattern characterized by increased Hp and Cp, altered serum protein fractions, and a reduced A/G ratio, whereas SAA was less consistent in the overall case–control comparison. The exploratory subgroup analyses suggest possible differences according to diagnostic profile, but they do not demonstrate diagnostic or prognostic performance of APPs. Future prospective studies, including clinically staged cats, longitudinal monitoring, and larger populations with complete paired serological and molecular testing, are warranted to clarify the clinical and possible prognostic value of APPs in feline leishmaniosis.

## 5. Conclusions

Cats positive for *L. infantum* antibodies and/or DNA showed a measurable inflammatory and dysproteinemic profile characterized by significantly increased Hp and Cp concentrations, higher total protein and globulin fractions, and a lower albumin concentration and A/G ratio compared with controls. In contrast, SAA did not differ significantly between groups, suggesting that this major APP may be less consistently altered when cats with heterogeneous diagnostic and clinical status are analyzed together.

Within *L. infantum*-positive cats, APPs were mainly associated with serum protein abnormalities, particularly albumin, the A/G ratio, and alpha2-, beta1-, and beta2-globulin fractions, whereas no significant association was found with the IFAT antibody titer. In exploratory analyses according to diagnostic profile, IFAT seropositivity was associated with higher Hp concentrations, whereas qPCR positivity was associated with higher SAA and Cp concentrations; however, these findings should be interpreted cautiously because qPCR-positive cats were few and qPCR positivity indicates parasite DNA detection rather than clinical disease activity. These findings support an integrated interpretation of IFAT, qPCR, APPs, and serum protein electrophoresis.

Overall, Hp and Cp may be useful to characterize the inflammatory profile of cats with laboratory evidence of *L. infantum* exposure and/or infection in the overall case–control setting. Assessment of APPs, particularly when combined with serum protein electrophoresis and both serological and molecular results, may contribute to the clinicopathological evaluation of cats infected with or exposed to *L. infantum*. However, their non-specific nature, possible confounding diseases, geographic heterogeneity, and lack of standardized clinical staging prevent direct attribution of these alterations to *L. infantum* alone. Further prospective and longitudinal studies in clinically well-characterized populations, with a higher number of PCR-tested and PCR-positive cats, are needed to clarify the clinical and possible prognostic value of these biomarkers in FeL.

## Figures and Tables

**Table 1:** Population characteristics of 63 healthy cats (controls) and 62 cats positive for *Leishmania infantum* IgG and/or DNA (cases). Significant *p*-values (<0.05) in bold.

Variable		Controls	Cases	*p*-Value
Lifestyle	Privately owned	31/63 (49.2%)	26/49 (53.1%)	0.552
	Stray colony	21/63 (33.3%)	18/49 (36.7%)	
	Shelter	11/63 (17.5%)	5/49 (10.2%)	
Origin	Lombardy	63/63 (100.0%)	20/62 (32.3%)	**<0.001**
	Sicily	0/63 (0.0%)	17/62 (27.4%)	-
	Istanbul (Turkey)	0/63 (0.0%)	23/62 (37.1%)	-
	Lazio	0/63 (0.0%)	2/62 (3.2%)	-
Breed	DSH/DLH	54/63 (85.7%)	39/45 (86.7%)	1.000
	Other breeds	9/63 (14.3%)	6/45 (13.3%)	
Gender	Male	30/63 (47.6%)	25/47 (53.2%)	0.700
	Female	33/63 (52.4%)	22/47 (46.8%)	
Reproductive status	Neutered	18/63 (28.6%)	12/24 (50.0%)	0.079
	Entire	45/63 (71.4%)	12/24 (50.0%)	
Age class	Kitten (<1 yr)	21/62 (33.9%)	4/42 (9.5%)	**0.004**
	Young adult (1–6 yrs)	30/62 (48.4%)	32/42 (76.2%)	**0.004**
	Mature adult (7–10 yrs)	4/62 (6.5%)	6/42 (14.3%)	0.185
	Senior (>10 yrs)	7/62 (11.3%)	0/42 (0.0%)	**0.024**

DSH: domestic shorthair cats, DLH: domestic longhair cat, yrs: years.

**Table 2:** Diagnostic profile of cats positive for *Leishmania infantum* IgG and/or DNA. The table summarizes the diagnostic criteria and distribution of IFAT and qPCR results.

Diagnostic Variable/Profile	n/Total (%)	Details
Cats tested by IFAT	62/62 (100.0)	IFAT cut-off ≥ 1:80
Cats tested by qPCR	34/62 (54.8)	22 blood samples; 12 lymph node samples
Positive only by IFAT	53/62 (85.5)	Most IFAT-seropositive cats had low titers
Positive only by qPCR	7/62 (11.3)	2 blood samples; 5 lymph node samples
Positive by both IFAT and qPCR	2/62 (3.2)	Both qPCR-positive samples were blood samples
IFAT-positive cats	55/62 (88.7)	Titers: 1:80, 44/55 (80.0%); 1:160, 8/55 (14.6%); 1:320, 1:1280, and 1:5120, each 1/55 (1.8%)
qPCR-positive cats	9/34 (26.5)	Median parasite burden 15 Leishmania/mL (range 5–250; IQR 13–65)

IFAT, indirect fluorescent antibody test; qPCR, real-time polymerase chain reaction.

**Table 3:** Comparison of selected acute phase proteins and protein profile between 63 healthy controls and 62 cats positive for *Leishmania infantum* IgG and/or DNA. Significant *p*-values (<0.05) in bold.

Variable	Healthy Controls Median (IQR)	*L. infantum*-Positive Cats Median (IQR)	*p*-Value
SAA (µg/mL; RI < 10)	0.63 (0.00–8.84)	0.00 (0.00–43.54)	0.187
Haptoglobin (mg/dL)	58.88 (41.17–78.81)	76.29 (43.59–139.92)	**0.013**
Ceruloplasmin (mg/dL)	16.13 (13.39–19.86)	18.54 (14.32–26.50)	**0.012**
Total protein (g/dL)	7.10 (6.70–7.70)	7.71 (6.98–8.52)	**0.002**
A/Gratio	1.20 (1.02–1.43)	0.95 (0.64–1.13)	**<0.001**
Albumin (g/dL)	3.88 (3.44–4.39)	3.58 (3.12–3.96)	**0.003**
Alpha1-globulin (g/dL)	0.18 (0.13–0.22)	0.15 (0.10–0.21)	0.075
Alpha2-globulin (g/dL)	1.30 (1.21–1.50)	1.19 (1.04–1.43)	**0.035**
Beta1-globulin (g/dL)	0.42 (0.36–0.48)	0.46 (0.37–0.60)	0.101
Beta2-globulin (g/dL)	0.23 (0.16–0.29)	0.35 (0.24–0.49)	**<0.001**
Gamma-globulin (g/dL)	1.02 (0.65–1.35)	1.58 (1.09–2.29)	**<0.001**

SAA: Serum amyloid A; IQR: interquartile range. A reference interval was available for SAA (<10 µg/mL). Validated feline reference intervals were not available for Hp and Cp with the assays used in this study.

**Table 4:** Exploratory analyses according to diagnostic test results in 63 healthy controls and 62 cats positive for *Leishmania infantum* IgG and/or DNA. Significant *p*-values (<0.05) in bold. Values are reported as median (interquartile range, IQR). Two-group comparisons were performed using the Mann–Whitney U test; four-subgroup comparisons were performed using the Kruskal–Wallis test.

Diagnostic Analysis	Group *p* Value	SAA (µg/mL)	Haptoglobin (mg/dL)	Ceruloplasmin (mg/dL)
IFAT serostatus	IFAT- (n = 70)	1.30 (0.00–12.06)	61.32 (40.62–82.54)	16.52 (13.63–20.25)
	IFAT+ (n = 55)	0.00 (0.00–41.95)	76.22 (44.19–143.40)	18.44 (14.15–26.49)
	*p*-value	0.733	**0.022**	0.093
qPCR status	qPCR- (n = 76)	0.73 (0.00–9.46)	59.42 (39.64–76.62)	16.52 (13.76–20.90)
	qPCR+ (n = 9)	30.76 (13.04–45.24)	77.78 (62.56–116.60)	24.68 (18.46–36.26)
	*p*-value	**0.001**	0.155	**0.004**
Combined IFAT/qPCR subgroups	IFAT-/qPCR- (n = 62)	0.72 (0.00–8.88)	59.42 (42.58–80.43)	16.17 (13.57–19.98)
	IFAT+/qPCR- (n = 14)	1.50 (0.00–11.57)	61.43 (36.20–76.00)	22.51 (15.41–26.70)
	IFAT-/qPCR+ (n = 7)	30.76 (11.22–38.87)	77.78 (50.97–102.23)	19.84 (18.44–26.68)
	IFAT+/qPCR+ (n = 2)	123.49 (69.08–177.91)	132.65 (97.61–167.69)	41.95 (39.10–44.80)
	*p*-value	**0.011**	0.488	**0.001**

IFAT, indirect immunofluorescent antibody test; IQR, interquartile range; qPCR, real-time polymerase chain reaction.

**Table 5:** Spearman’s correlations between acute phase proteins and protein profile variables in 62 cats positive for *Leishmania infantum* IgG and/or DNA. Significant *p*-values (<0.05) in bold.

Variable	SAA	Haptoglobin	Ceruloplasmin
Age (yrs)	−0.049 (*p* = 0.756)	−0.158 (*p* = 0.318)	0.038 (*p* = 0.813)
Total protein (g/dL)	−0.093 (*p* = 0.473)	0.137 (*p* = 0.288)	**0.268 (*p* = 0.035)**
Albumin-to-globulin ratio	−0.191 (*p* = 0.137)	**−0.392 (*p* = 0.002)**	**−0.326 (*p* = 0.010)**
Albumin (g/dL)	**−0.353 (*p* = 0.005)**	**−0.440 (*p* < 0.001)**	−0.162 (*p* = 0.208)
Alpha1-globulin (g/dL)	−0.040 (*p* = 0.759)	−0.211 (*p* = 0.099)	0.124 (*p* = 0.336)
Alpha2-globulin (g/dL)	**0.447 (*p* < 0.001)**	**0.492 (*p* < 0.001)**	0.176 (*p* = 0.171)
Beta1-globulin (g/dL)	0.175 (*p* = 0.175)	0.125 (*p* = 0.331)	**0.342 (*p* = 0.007)**
Beta2-globulin (g/dL)	0.020 (*p* = 0.876)	0.004 (*p* = 0.973)	**0.256 (*p* = 0.045)**
Gamma-globulin (g/dL)	−0.090 (*p* = 0.486)	0.236 (*p* = 0.065)	0.169 (*p* = 0.188)
IFAT antibody titer	0.028 (*p* = 0.842)	0.214 (*p* = 0.117)	0.095 (*p* = 0.490)

SAA: Serum amyloid A; IFAT: indirect immunofluorescence antibody test.

## Data Availability

All original data and contributions generated in this study are reported within the article. Any additional information or requests may be addressed to the corresponding authors (Germano Castelli, Federica Bruno).

## Abbreviations

The following abbreviations are used in this manuscript:

APP	Acute phase proteins
Cp	Ceruloplasmin
FeL	Feline leishmaniosis
FeLV	Feline leukemia virus
FIV	Feline immunodeficiency virus
Hp	Haptoglobin
IFAT	Indirect immunofluorescence antibody test
qPCR	Real-time polymerase chain reaction
SAA	Serum amyloid A
